# Time-resolved luminescence and excitation spectroscopy of Co-doped Gd_3_Ga_3_Al_2_O_12_ scintillating crystals

**DOI:** 10.1038/s41598-020-77451-x

**Published:** 2020-11-23

**Authors:** Viktorija Pankratova, Anna P. Kozlova, Oleg A. Buzanov, Kirill Chernenko, Roman Shendrik, Anatolijs Šarakovskis, Vladimir Pankratov

**Affiliations:** 1 Institute of Solid State Physics, University of Latvia, 8 Kengaraga iela, Riga, LV-1063 Latvia; 2 National University of Science and Technology “MISiS”, Leninsky Prospekt 4, 119049 Moscow, Russia; 3 OJSC ‘Fomos-Materials’ Co, Buzheninova street 16, 107023 Moscow, Russia; 4 MAX IV Laboratory, Lund University, PO BOX 118, 221 00 Lund, Sweden; 5 Vinogradov Institute of Geochemistry, SB RAS, 1a Favorskii Street, 664033 Irkutsk, Russia

**Keywords:** Materials science, Optical spectroscopy

## Abstract

Cerium doped Gd_3_Ga_3_Al_2_O_12_ (GGAG) single crystals as well as GGAG:Ce single crystals co-doped by divalent (Mg^2+^, Ca^2+^) and tetravalent (Zr^4+^, Ti^4+^) ions have been studied by means of time-resolved luminescence as well as the excitation luminescence spectroscopy in vacuum ultraviolet (VUV) and soft X-ray (XUV) spectral range. Tunable laser excitation was applied for time-resolved experiments in order to obtain luminescence decay curves under excitations in Ce^3+^, Gd^3+^ and excitonic absorption bands. The influence of the co-dopant ions on the Ce^3+^ luminescence decay kinetics is elucidated. The fastest luminescence decay was observed for the Mg^2+^ co-doped crystals under any excitation below bandgap energy indicating the perturbation of the *5d* states of Ce^3+^ by Mg^2+^ ions. Synchrotron radiation was utilized for the luminescence excitation in the energy range from 4.5 to 800 eV. Special attention was paid to the analysis of Ce^3+^ excitation spectra in VUV and XUV spectral range where multiplication of electronic excitation (MEE) processes occur. Our results demonstrated that GGAG:Ce single crystals co-doped by Mg^2+^ ions as well as the GGAG:Ce crystal annealed in vacuum reveal the most efficient excitation of Ce^3+^ emission in VUV-XUV excitation range. The role of intrinsic defects in MEE processes in the co-doped as well as in the annealed GGAG:Ce single crystals is discussed.

## Introduction

Nowadays scintillator detectors play an irreplaceable role in high-energy physics, safety systems, space and medical applications^1–5^. Among other scintillator materials^1,2,6–8^ cerium doped gallium gadolinium aluminum garnet (Gd_3_Ga_3_Al_2_O_12_:Ce or GGAG:Ce) nowadays is one of the most relevant scintillator materials because it comprises the combination of such properties, as a very high light output of scintillations (58,000 photons/MeV)^1^, high density (6.63 g/cm^3^) and relatively fast decay time (90 ns)^9–11^. Furthermore, this material is very suitable for neutron detection and discrimination, thanks to the high Gd cross-section for neutron interaction^12,13^. However, the practical application of this compound is restricted by long afterglow of Ce^3+^ emission, which is explained by the intermediate localization of electrons and holes at shallow traps during energy transfers to the Ce^3+^ emission centers. One of possible ways to accelerate Ce^3+^ emission decay is the co-doping of the GGAG:Ce single crystals by divalent ions^10,14–16^. In this case, that lack of positive charge induced by divalent ion is compensated by changing the valence of cerium ions from 3 + to 4 + . To create a luminescence center in the form of an excited Ce^3+^ center, the Ce^4+^ ion must capture an electron from the conduction band. This means that the processes of energy transfer to emission centers do not include the hole trapping stage, which is necessary in the case of the Ce^3+^ ion. As a result, radiative relaxation process is significantly accelerated in divalent co-doped crystals^14,17^. However, this approach has a serious drawback. The co-doping of the GGAG:Ce crystals by divalent ions strongly quenches the scintillation light yield, which is preliminarily explained by the shift of *5d* states of Ce^3+^ towards the bottom of the conduction band as well as due to the creation of the deep traps originated from co-dopant ions. In contrast to divalent ions the effect of co-doping with tetravalent cation has not been sufficiently studied for GGAG:Ce. It is only known that the investigation of Hf^4+^ doped GGAG:Ce demonstrates the degradation of the light output and longer decay time compared with the conventional GGAG:Ce^18^. On the other hand, Zr^4+^ co-doped GSO:Ce has shown 20% higher light output in respect of non-co-doped analogue^19^.

Therefore, the influence of co-dopant ions on the luminescence properties of GGAG:Ce single crystals must be studied tuning the excitation energy which covers the ranges of intrinsic *4f–5d* transitions of Ce^3+^ ion, excitonic and band-to-band transitions. Thus, in current work we have applied the excitations produced by the tunable laser excitations (in 210–420 nm range) in order to compare time-resolved luminescence characteristics under excitations in Ce^3+^, Gd^3+^, defect and excitonic absorption bands of GGAG:Ce single crystals co-doped by divalent and tetravalent ions.

The improvement of energy and time resolution of scintillating materials is of a great concern for the breakthrough in many practical applications. Both of these critical parameters improve if scintillation light yield increases. Therefore, the number of electron–hole pairs generated in a scintillator by an arriving photon/particle defines the energy and time resolutions. Thus, the understanding of the multiplications of electronic excitations processes in GGAG:Ce is necessary, which have been poorly studied so far in this material. Moreover, in the case of doped materials, electrons can multiply the excitation by impact ionization of the impurities if the mean free path of the fast electron is long enough to encounter an impurity ion. The GGAG compound belongs to the class of wide band gap materials having the band gap about 6.2 eV. This energy and higher refers to the vacuum ultraviolet (VUV) and soft X-ray (XUV) spectral range. Luminescence excitation spectra of wide bandgap materials in the VUV region provide important information about the last stage of the cascade process of the electronic excitation multiplication^20^. In addition, the excitation spectra in wide spectral range allow to estimate the migration energy losses using the correlation between intensities of direct excitation of Ce^3+^ ions and the excitation via the bulk in the fundamental region. The most suitable excitation source in VUV-XUV range is synchrotron radiation, which was successfully utilized for the excitation spectroscopy of wide band gap materials^21–28^ and semiconductors as well^29–31^.

The tuneability of synchrotron radiation allows to excite samples by high-energy photons, which corresponds to the energy of hot charge carriers as well as to the energy when multiplication of electronic excitation processes are occurring. In addition, surface quality of the scintillator plays an important role in the photoelectron production efficiency due to a low penetration depth of VUV photons into crystals. The absorption coefficient close to the fundamental absorption region is about 10^6^ cm^−1^ and excitation penetration is several tens of nanometers^20^. The electronic excitations generated by VUV quanta may migrate to the surface and not take part in the bulk recombination process. They may recombine at the surface (radiatively or non-radiatively) or escape from the crystal (electron emission). Consequently, understanding of physical mechanisms related to the excitation energy absorption and scattering in a close-to-surface layer and surface area of scintillator crystals is essential. With the increase of the excitation energy, the penetration depth of incident photons becomes larger and it is supposed that only bulk states are excited under XUV photons. Therefore, varying the excitation energy in VUV-XUV range one can manipulate the penetration depth of photons and can distinguish surface and bulk related relaxation processes.

The luminescence yield depends also on the quality of the crystal. The presence of absorbing and scattering centers in the crystal is a common source of scintillation losses^1^. A post growth annealing of the samples leads to alteration of concentration of point defects in crystals. The most essential changes during annealing occur, however, on the crystals’ surfaces affecting surface loss centers. Taking into account that VUV excitations are very sensitive in respect of surface states it is expected that the crystals with distinguished surface quality may reveal the nature of surface loss centers. To our knowledge VUV excitation spectroscopy data for annealed GGAG:Ce single crystals have not been reported yet.

In the current paper we report time-resolved luminescence and VUV and XUV excitation spectroscopy of co-doped GGAG:Ce single crystals as well as the GGAG:Ce crystals annealed in different atmospheres. The origins of the modification of energy transfer efficiency to the emission center in the co-doped as well as annealed GGAG:Ce single crystals are discussed in the paper. Furthermore, the influence of co-dopant ions on the temporal characteristics of luminescence centers in GGAG:Ce crystals will be elucidated.

## Method

All GGAG single crystals studied in current research have been grown by Czochralski method in JSC “Fomos-Materials” (Moscow, Russia). The details of crystals’ growth of doped and co-doped GGAG single crystals have been reported in^32^. The crystals have been cut in small thin pieces and polished for optical experiments. The cerium concentration in the doped crystals was 3 at%. The concentrations of the co-dopants in the GGAG:Ce single crystals as well samples thicknesses are summarized in Table 1. As one can see from the Table 1, two GGAG:Ce are co-doped by divalent ions (Mg^2+^ or Ca^2+^) which induce Ce^4+^ centers in the corresponding crystals. One crystal was co-doped by Zr^4+^ ions to test the hypothesis expressed in^19^ that tetravalent ions can increase luminescence light output. The co-doping of one of GGAG:Ce,Mg single crystals by Ti^4+^ was justified by the restoration of the luminescence light yield which was deteriorated due to the Mg^2+^ co-dopants. In addition, the GGAG:Ce single crystal were post annealed in vacuum or in oxygen atmosphere. The annealing details please see in Table 1.

**Table 1:** The GGAG single crystals studied in current research.

Sample	Dopants and their concentrations	Sample thickness, mm	Annealing
GGAG	Undoped	2.04	–
GGAG:Ce	Ce^3+^ (3 at%)	0.49	–
			Vacuum (10^–5^ torr) for 30 min at 1000 °C
			Oxygen atmosphere (pressure 1.2 atm) for 10 h at 1000 °C
GGAG:Ce,Zr	Ce^3+^ (3 at%) Zr^4+^ (100 ppm)	2.20	–
GGAG:Ce,Mg	Ce^3+^ (3 at%) Mg^2+^ (350 ppm)	1.92	–
GGAG:Ce,Mg,Ti	Ce^3+^ (3 at%) Mg^2+^ (80 ppm) Ti^4+^ (30 ppm)	1.92	–
GGAG:Ce,Ca	Ce^3+^ (3 at%) Ca^2+^ (150 ppm)	1.68	–

Optical properties of the crystals at room temperature have been studied by means of the Cary 7000 optical spectrophotometer. The low temperature absorption spectra have been obtained in the Perkin-Elmer Lambda 950 UV/VIS/NIR spectrophotometer at 7 K using cryocooler Janis Research CCS-100/204N. Time-resolved luminescence spectra were obtained upon excitation by wavelength-tunable pulsed solid-state laser Ekspla NT342/3UV (210–2300 nm). The emission signal was detected by the Andor iSTAR DH734-18 mm CCD camera coupled to the Andor SR-303i-B spectrometer. Luminescence decay kinetics were measured by a photomultiplier tube PG122 (time resolution better than 5 ns) and digital oscilloscope Tektronix TDS 684A.

A luminescence excitation spectroscopy experiments have been carried out under synchrotron radiation excitation. These experiments have been performed on the photoluminescence endstation *FINESTLUMI*^33,34^ of the FinEstBeAMS undulator beamline^35^, which was constructed and developed at 1.5 GeV storage ring of MAX IV synchrotron facility (Lund, Sweden). The excitation energy range was 4.5–800 eV, while temperature varied from 10 to 300 K. The excitation spectra were normalized utilizing the calibration curve obtained by means of AXUV-100G diode. Luminescence detection in UV–visible spectral range (200–800 nm) was performed by the Andor Shamrock (SR-303i) 0.3 m spectrometer having the grating (300/500). The Andor Shamrock spectrometer was equipped with the photomultiplier photon counting heads (H8259-01 Hamamatsu) covering the spectral range from 200 to 900 nm.

## Results and discussion

### Absorption spectra

**Figure 1:**
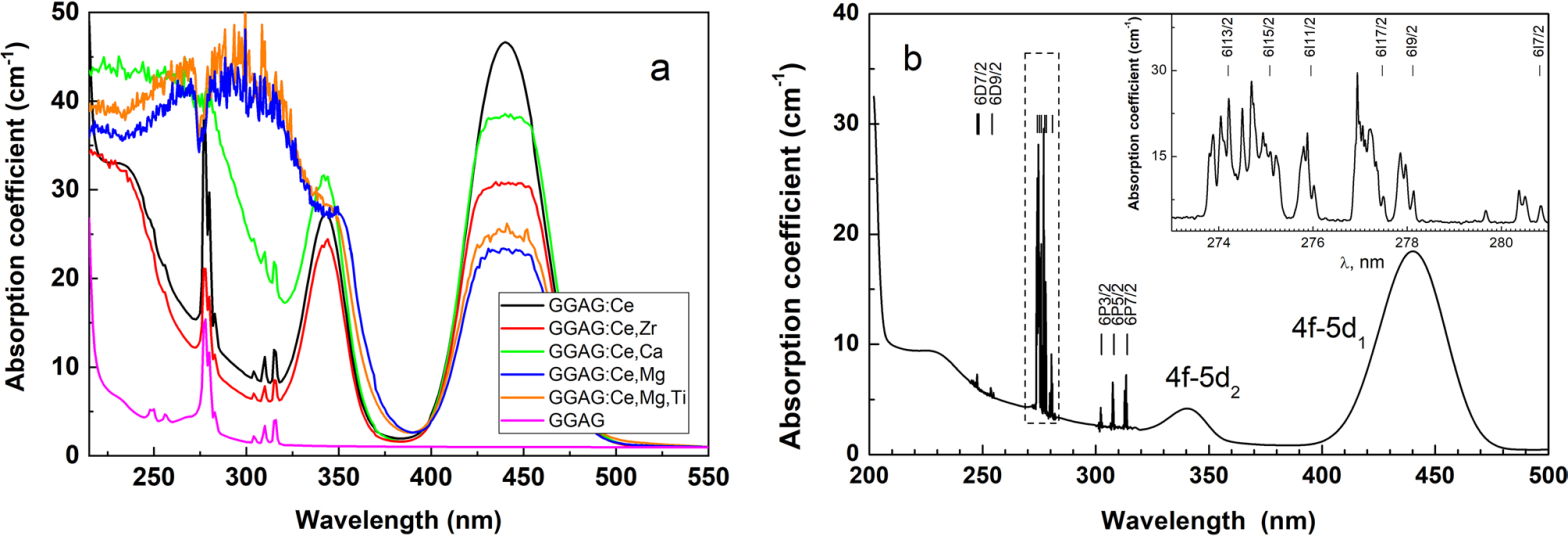
The absorption spectra of the co-doped GGAG:Ce single crystals at room temperature (**a**). The spectrum for the undoped crystal is given for comparison. The absorption spectrum for the cerium doped GGAG single crystal at 10 K is shown in (**b**). The inset reveals a fine structure of Gd^3+^ transitions in the dashed area. Gd^3+^ absorption lines are assigned to energy levels reported in^40^.

The Fig. 1a demonstrates the absorption spectra of all GGAG crystals studied at room temperature. The low temperature absorption spectrum for the cerium doped GGAG single crystal is shown in Fig. 1b. The absorption spectrum for the undoped GGAG (Fig. 1a) reveals sharp absorption lines due to *f–f* transitions in Gd^3+^ ions. These lines can be observed also in doped and co-doped crystals. The absorption spectrum for the GGAG:Ce (Fig. 1a) contains two broad bands at 440 nm (*4f–5d*_*1*_) and 343 nm (*4f1–5d*_*2*_) due to *4f–5d* transitions in Ce^3+^. In addition, the broad absorption band below 270 nm appears in the spectrum for GGAG:Ce if one compares the spectrum for the undoped crystal (Fig. 1a). This band is located below the fundamental absorption edge and it is observed in all cerium doped crystals. However, in the Ca^2+^ co-doped crystal and especially in the Mg^2+^ co-doped crystals the intensity of absorption in the UV spectral range (below 300) nm is huge. The optical density this absorption band is high and, therefore, the shape of this band is flawed and much thinner crystals should be measured. The same could be also said about the absorption band of *4f–5d*_*1*_ transitions, which looks truncated for all co-doped crystals except the most thinner GGAG:Ce crystal. Nevertheless, some preliminary conclusions can be made based on the spectra depicted in Fig. 1a. Obviously, the strong UV absorption observed for the Ca^2+^ and Mg^2+^ co-doped crystals is induced by co-dopant ions. Substituting Gd^3+^ ion in GGAG lattice Ca^2+^ and Mg^2+^ ions give extra negative charge, which should be compensated. The charge compensated centers can be vacancies or O^-^ oxygen deficit centers well known in complex oxides^36–38^. However, the most probable compensation is the partial conversion of Ce^3+^ to Ce^4+^, which is confirmed by XANES experiments^16^. Therefore, the intensive UV absorption in the absorption spectra for the Ca^2+^ and Mg^2+^ co-doped crystals is explained by the increased concentration of Ce^4+^ ions^16,17,39^, namely due to charge transfer transition from O^2-^ to Ce^4+^ center. On the other hand, the absorption spectrum for the Zr^4+^ co-doped crystal is almost the same as the spectrum for GGAG:Ce. In the case of Zr^4+^ substitution of Gd^3+^ ion one extra positive charge is introduced to the GGAG lattice. The charge compensator in this case is questionable. However, Zr^4+^ should eliminate any residual Ce^4+^ ions in the crystal and, obviously, the absorption band (below 270 nm) in the spectra for GGAG:Ce and for Zr^4+^ co-doped crystals is not due to Ce^4+^ center. Furthermore, the strong UV absorption in the spectra for the Ca^2+^ and Mg^2+^ co-doped crystals is too wide and, perhaps, contains several overlapped absorption bands. Another suggestion is that the different absorption centers are responsible for the UV absorption bands observed in the GGAG:Ce and the Zr^4+^ co-doped crystals from one side and the Ca^2+^ and the Mg^2+^ co-doped crystal from another one. We will return to this discussion in the following sections considering the excitation spectra of Ce^3+^ emission.

### Luminescence spectra

**Figure 2:**
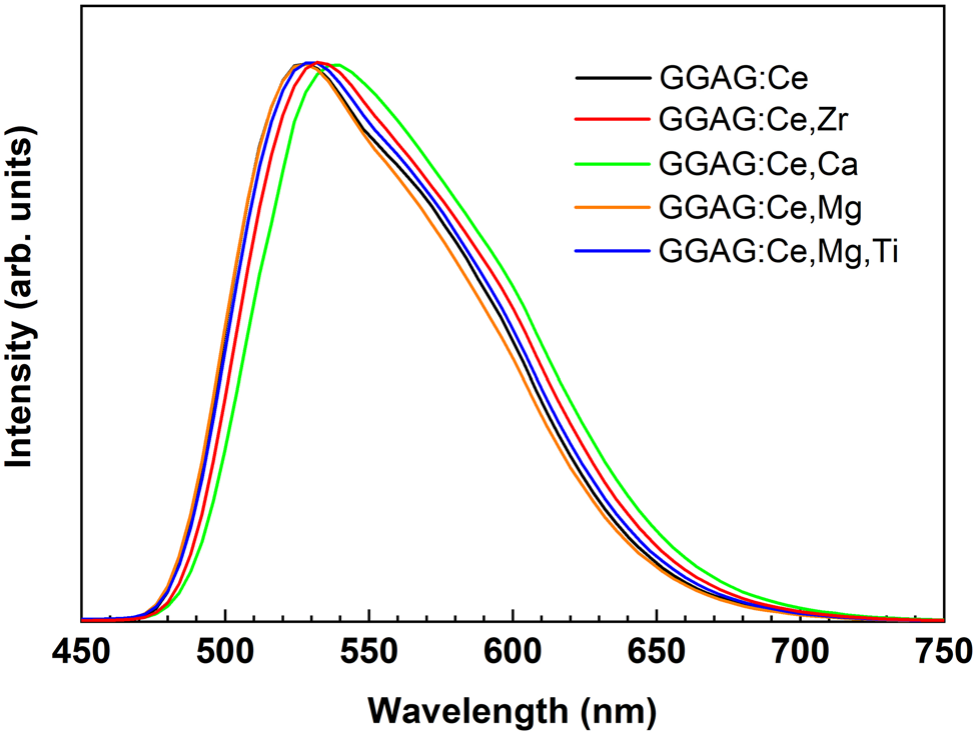
The luminescence spectra of the GGAG:Ce as well as of the GGAG:Ce co-doped single crystals under 45 eV excitation at 10 K. The spectra are normalized at maximum intensity of Ce^3+^ emission band.

The emission spectra of Ce^3+^ emission in the GGAG:Ce single crystal and in the GGAG:Ce co-doped crystals at low temperature under deep VUV (45 eV) excitation are shown in Fig. 2. The spectra are normalized at the maximum intensity of the emission bands. One can see (Fig. 2) that the co-dopant ions influence the shape of Ce^3+^ emission bands. The Ca^2+^ ions induce the most essential changes shifting the Ce^3+^ emission band towards the long wavelength spectral side comparing with the corresponding spectrum of GGAG:Ce crystal. On the other hand, the emission spectrum in the Mg^2+^ co-doped crystal well coincides with the corresponding spectrum observed in the crystal without co-dopants. The origin of the observed changes in the emission spectra can be connected with the alteration of Al/Ga ratio in the co-doped crystals. It means that one can manipulate the band gap value of this compound tuning the stoichiometry of GGAG:Ce as well as to adjust Ce^3+^ levels in the forbidden gap^41,42^. It was established before that the red shifting of the Ce^3+^ luminescence bands takes place if the ratio of the Al/Ga increases^43^. Thus, the influence of the Ca^2+^ and Zr^4+^ ions on the Al/Ga ratio explains the red shift of the emission spectra in the corresponding crystals depicted in Fig. 2. Another possible explanation is the perturbation of some Ce^3+^ states by co-dopant ions as it was recently suggested^44^. It is assumed that the strongest perturbation is induced by ions with biggest ionic radii among co-dopants, i.e. Ca^2+^ and Zr^4+^. The Ce^3+^ emission results from 5d–4f radiation transition. Taking into account that the 4f ground state of Ce^3+^ is split into two levels (^2^F_7/2_ and ^2^F_5/2_) each luminescence spectrum (in Fig. 2) is represented by two overlapped emission bands due to 5d-^2^F_7/2_ and 5d-^2^F_5/2_ transitions. The perturbation can lead not only to the red shift of the emission spectra towards low energy side but also can change the ratio of 5d-^2^F_7/2_ and 5d-^2^F_5/2_ transitions leading to the more pronounced low energy shoulder in the emission spectra of the Ca^2+^ and Zr^4+^ co-doped crystals.

### Excitation spectra

**Figure 3:**
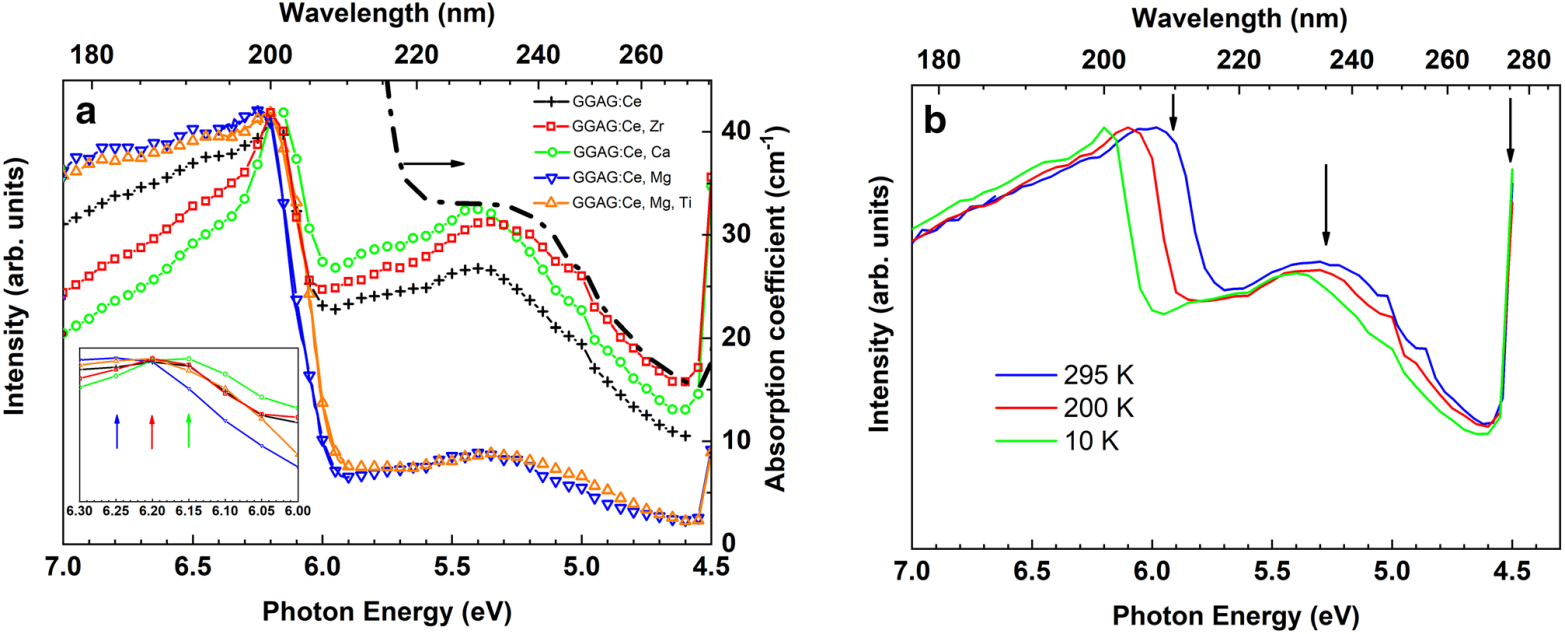
The low energy excitation spectra of Ce^3+^ emission (530 nm) in the co-doped GGAG:Ce crystals at 10 K. Dashed line shows the part of the absorption spectrum for the GGAG:Ce single crystal taken from Fig. 1 for the comparison (**a**). Inset (**a**) exhibits the excitonic range of the excitation spectra in details where the colored arrows indicate the position of the corresponding peak (see details in the text). The temperature dependence of the excitation spectra of Ce^3+^ emission (530 nm) in the GGAG:Ce crystal (**b**). The arrows show the excitation energies utilized in the time-resolved experiments (see details in the text).

The excitation spectra in the 4.7–7.0 eV excitation region of Ce^3+^ emission are shown in Fig. 3a for all crystals studied at 10 K, while the temperature dependency of the excitation spectra in GGAG:Ce single crystal is depicted in Fig. 3b. Considering these excitation spectra (Fig. 3a, inset) one can see that the excitonic peak position at about 200 nm or 6.2 eV is co-dopant dependent. The excitonic peak for the Ca^2+^ co-doped crystals slightly red shifted in respect to the excitonic peaks observed for other crystals peaking at 6.15 eV. The excitonic peaks observed for GGAG:Ce, Zr^4+^ co-doped and Mg + Ti co-doped crystals are similar and have maximum at 6.2 eV. The excitation spectra of Mg^2+^ co-doped crystals are slightly blue shifted with respect of others Ce^3+^ excitation spectra peaking at 6.25 eV. Analyzing the excitation spectra in the spectral range close to the excitonic transitions, we can suggest that co-doped ions can slightly modify the stoichiometry of the crystals. In fact, one can slightly change the Al/Ga ratio as well as the content of Gd^3+^ in the lattice by introducing a co-dopant ion into the GGAG lattice. Based on the concept of the band gap engineering in garnets^41,45^ the band gap value can be manipulated tuning the Al/Ga ratio in the GGAG. The alterations in the band gap values lead to shifts of the excitonic bands as depicted in Fig. 3a as well as to the shifts of the emission bands (Fig. 2). Indeed, the emission and excitation spectra observed in Figs. 2 and 3a for the Ca co-doped crystal are slightly red shifted in respect to others. On the other hand, the spectra obtained for the Mg co-doped crystal are blue shifted.

Figure 3b demonstrates the temperature shift of the excitonic peak in the excitation spectrum of Ce^3+^ emission in the GGAG:Ce single crystal. The temperature behavior of the corresponding spectra observed in the co-doped crystals is similar. Figure 3b demonstrates the blue shift of excitonic peak from 210 to 200 nm decreasing the sample temperature from 295 to 10 K. By analogy with other garnets (YAG, LuAG, etc.) we assume that exciton in GGAG exists in form of a self-trapped state. Therefore, a strong electron phonon coupling takes place and a self-trapped exciton (STE) can be considered as localized center. A temperature behavior of the excitonic peak (Fig. 3b) is typical for STE. The temperature shift of the excitonic peak allows us to apply the 210 nm laser excitation at room temperature for the study of the Ce^3+^ decay kinetics under direct excitation in the excitonic band (please see the “Time-resolved luminescence”).

Next, we are focusing on the low energy part of the excitation spectra in Fig. 3a. Each excitation spectrum contains the wide excitation band at energies below excitonic transitions (4.5–6.0 eV range peaking at about 5.3 eV). These excitation bands are similar to the high-energy absorption band observed in the absorption spectra (Fig. 1a) of the GGAG:Ce and the Zr^4+^ co-doped crystals shown by the dashed line in Fig. 3a for comparison. This absorption band was not resolved in the absorption spectra for the Ca^2+^, Mg^2+^ and Mg + Ti crystals due to the overlapping with the strong absorption of Ce^4+^ band. As we already mentioned above the absorption of Ce^4+^ is due to electron transfer from the valence band to the ground state of Ce^4+^ forming Ce^3+^ in ground state. It means that a direct excitation in Ce^4+^ absorption band does not lead to Ce^3+^ emission and, therefore, the Ce^4+^ absorption cannot be revealed in the excitation spectra in Fig. 3a. This result confirms our suggestion that the strong absorption in UV range of the absorption spectra (Fig. 1a) contains at least two overlapped bands: the charge transfer band O^2−^—Ce^4+^ and the band peaking about 5.3 eV.

The origin of the 5.3 eV (or UV) band observed in the absorption and the excitation spectra needs further comments. Based on our result we can suggest that the UV band is due to transitions from the Ce^3+^ ground state to the *5d*_*3*_ excited state. This suggestion explains why the UV band appears in the absorption spectra of the Ce^3+^ doped GGAG crystals (Fig. 1) but it is absent in the spectrum for the undoped GGAG. Furthermore, the intensity of the UV excitation band is three times less in both magnesium containing co-doped crystals if to compare with other single crystals studied (Fig. 3a). It means that the concentration of Ce^3+^ ions is smaller in the magnesium co-doped crystals despite the equal cerium concentration in the furnace charges. However, some part of Ce^3+^ ions were transformed to the Ce^4+^ ions in order to compensate the lack of the positive charge due to Mg^2+^ co-dopant ions. The existence of the Ce^4+^ ions in the Mg^2+^ co-doped crystals was confirmed by our recent XANES experiments^44^. These results also demonstrated that all other crystals, including the Ca^2+^ co-doped one, do not reveal the existence of Ce^4+^ ions. Therefore, the Ce^3+^ concentration should be more or less the same therein. Hence, the intensity of UV excitation band should be similar in the spectra for all crystals excluding the Mg^2+^ co-doped ones, as one can see in Fig. 3a. Nevertheless, the strong decreasing of the UV excitation band (Fig. 3a) cannot be explained only by a simple concentration decreasing of Ce^3+^ ions in the Mg^2+^ co-doped crystals. In addition, the spectral position of the UV absorption band, which we explained as *4f–5d*_*3*_ absorption in Ce^3+^ and the Ce^4+^ absorption band are very close, i.e. these band are spectrally overlapping. Therefore, these two centers in the Mg^2+^ co-doped crystals compete with each other absorbing photons in UV spectral range. It means that UV photons instead of being absorbed by Ce^3+^ ion are absorbed by Ce^4+^ forming Ce^3+^ in the ground state and do not participate in the luminescence process. It also should be noted that our conclusion about the origin of the 5.3 eV excitation band can be indirectly supported by the theoretically re-assignment of Ce^3+^ absorption transitions in YAG reported in^46^.

**Figure 4:**
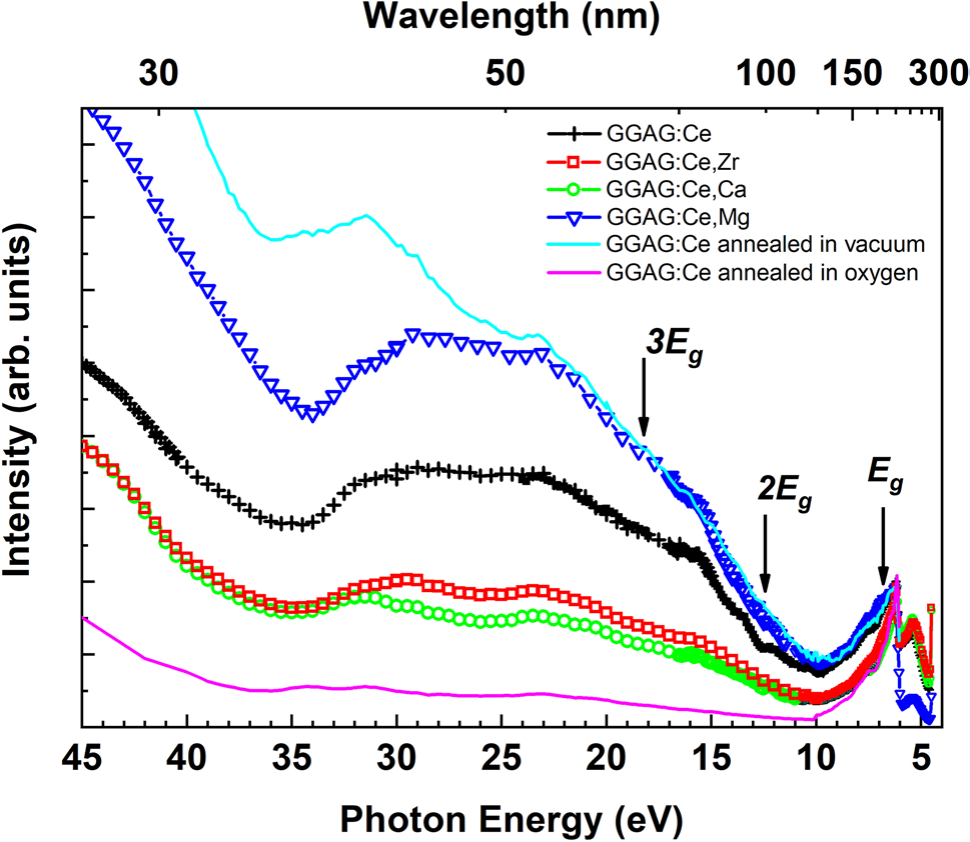
The excitation spectra of Ce^3+^ emission (530 nm) in VUV spectral range measured at 10 K for the co-doped GGAG:Ce single crystals as well as for the annealed crystals. All spectra are normalized to the excitonic band.

The excitation spectra of Ce^3+^ emission in the crystals studied have also significant discrepancies in the VUV spectral range. Figure 4 demonstrates in fact the extension of the excitation spectra in Fig. 3a up to 45 eV. For each excitation spectrum there is the excitation intensity degradation at energies just above the energy fundamental transitions (~ 6.5 eV) reaching minimum value at about 10 eV. Thereafter, the excitation intensity starts to rise up at energies higher than 12–13 eV in all excitation spectra observed in Fig. 4. This energy is roughly equal to the value of *2E*_*g*_, where *E*_*g*_ is a band gap energy in GGAG. Such intensity behavior means that so called multiplication of electronic excitations processes are observed. The occurrence of MEE processes means that two or more emission centers are excited per one absorbed photon during inelastic electro-electron scattering. In order to realize successfully the MEE processes, the excitation energy of the incident photon must be higher than the threshold energy, which is 2*E*_*g*_^47^. If energy of hot electrons is much higher than the threshold energy (3*E*_*g*,_ 4*E*_*g*_, etc.), more secondary electrons can be generated and subsequently more luminescence centers can be excited. All spectra in Fig. 4 have similar peculiarities in the spectral range 10–45 eV except the excitation intensity, which distinguishes for different crystals. It implies that the efficiency of MEE processes is sample dependent. One of the possible explanations is following. All single crystals studied contain some amount of intrinsic defects. We suggest that these defects can be efficient traps for hot charge carriers, which leads to the diminishing of MEE processes, and, subsequently plays a negative role in scintillation performing. The best excitation efficiency in VUV spectral range demonstrates the Mg^2+^ co-doped crystal. It is known that the Mg^2+^ co-doping of GGAG:Ce crystals leads to the effective elimination of shallow electron traps due to oxygen vacancies or gallium deficiency^48^. In particular, as it was assumed in^48^ the Mg^2+^ co-doping eliminates gallium vacancies from Ce:GGAG crystals when Mg^2+^ ions occupy the sites of gallium vacancies. This is essentially the same as the case for oxygen vacancies. On the other hand, the Zr^4+^ and Ca^2+^ co-doped crystals, perhaps, have some extra centers for non-radiative relaxation of hot charge carriers. We can suppose that such centers can be charge compensators of extra positive charge induced by Zr^4+^ ions in the Zr^4+^ co-doped crystal. The low efficiency of MEE processes in the Ca^2+^ doped crystal is not clear and further studies are needed. We assume, for example, that a comparison of the excitation spectra of GGAG:Ce crystals with different concentrations of Ca^2+^ ions could reveal this ambiguity.

**Figure 5:**
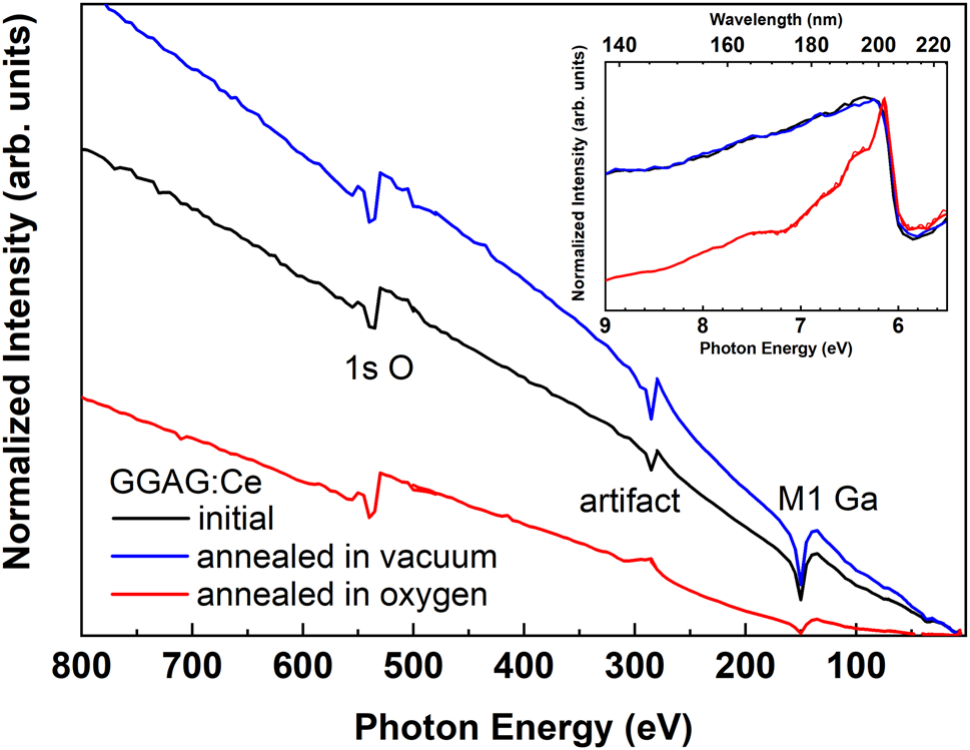
The low temperature excitation spectra of Ce^3+^ emission (530 nm) in VUV-XUV spectral range for the annealed GGAG:Ce single crystals. All spectra are normalized to the excitonic band.

The importance of lattice defects and their influences on the MEE processes are clearly demonstrated by the next example. Figures 4 and 5 show the comparison of the excitation spectra of Ce^3+^ emission in the GGAG:Ce single crystal annealed in vacuum and oxygen atmosphere. An absorption coefficient is huge for excitation energy exceeding bandgap energy; therefore, VUV photons are absorbed in very thin surface layer. On the other hand, the penetration depth of the X-rays is much deeper exciting the luminescence centers in samples’ bulk. The penetration depth of VUV and XUV photons can be estimated as the attenuation length of incident photons i.e. the depth into the material measured along the surface normal where the intensity of photons falls to *1/e* of its value at the surface. Based on the approach described in details in^49,50^ the calculated attenuation length of VUV and soft X-ray photons in the energy range from 30 to 1000 eV falling on the GGAG (g = 6.63 g/cm^3^) is obtained (Fig. S1 of supplementary materials). This result demonstrates that the attenuation lengths is about 10 nm of 30 eV photons, while it reaches 0.5 microns if photon energy increases up to 1000 eV.

The excitation spectra depicted in Fig. 5 are measured in very wide spectral range 5.5–800 eV covering regions of both VUV and soft X-rays photons. By analogy with other excitation spectra (Figs. 3 and 4), the spectra in Fig. 5 are also normalized in the excitonic band. The labels *1s O* and *M1 Ga* in Fig. 5 correspond to the absorption energies by *1s* core level of oxygen and *M1* edge of gallium, respectively. The inset of Fig. 5 clearly shows that the excitation spectra at low energies for the initial and vacuum annealed crystals are identical, while the excitation intensity decrease in the spectrum of the crystal annealed in oxygen is observed at energies exceeding excitonic energy. This strong intensity drop can be explained by an efficient trapping of charge carriers by non-radiative centers (defects) in the crystal annealed in oxygen. As we mentioned above, in VUV spectral range photons are mostly absorbed in thin surface layer of the crystal. Thus, we suggest that the strong degradation of the luminescence under excitation by VUV photons can be explained by surface defects induced by thermal treatment in oxygen. However, passing from VUV to soft X-ray range where the influence of surface is negligible the intensity of luminescence remains significantly smaller in the annealed in oxygen crystal. It means that the annealing in oxygen induces non-radiative centers (defects) in crystals bulk. The origin of these defects is unclear. Obviously, the concentration of oxygen vacancies cannot increase after annealing in oxygen atmosphere. We assume that the effect of annealing under oxygen pressure can be explained by the violation of stoichiometry in the surface layer. On one hand, some of Ga ions are accommodated at interstitial sites, e.g. the *b* sites of the garnet structure similarly to GGG crystals^51^. On the other hand, some Ga_2_O_3_ evaporates from the surface during annealing at temperatures higher than 1100 °C^51,52^. The non-stoichiometry layer depth could be increased up to several micrometers during the ten hours annealing. Therefore, the stoichiometry of the 10 h annealed crystals differs if compared with unannealed samples. GGAG single crystals having slight excess of Ga demonstrated a lower proportionality than the stoichiometric ones^42^. In addition, MEE could be less efficient in the non-stoichiometry samples due to defect levels induced by non-stoichiometry.

In contrast to the annealing in oxygen atmosphere, oxygen vacancies can be created after annealing in vacuum. However, the excitation spectrum reveals higher luminescence intensity under high-energy excitation than the corresponding spectrum for the initial crystal. It means that annealing in vacuum improves luminescence properties eliminating quenching centers in GGAG:Ce single crystal. The origin of the defects induced or eliminated by annealing in oxygen atmosphere or vacuum should be clarified and further studies are needed varying annealing temperature and time.

### Time-resolved luminescence

**Figure 6:**
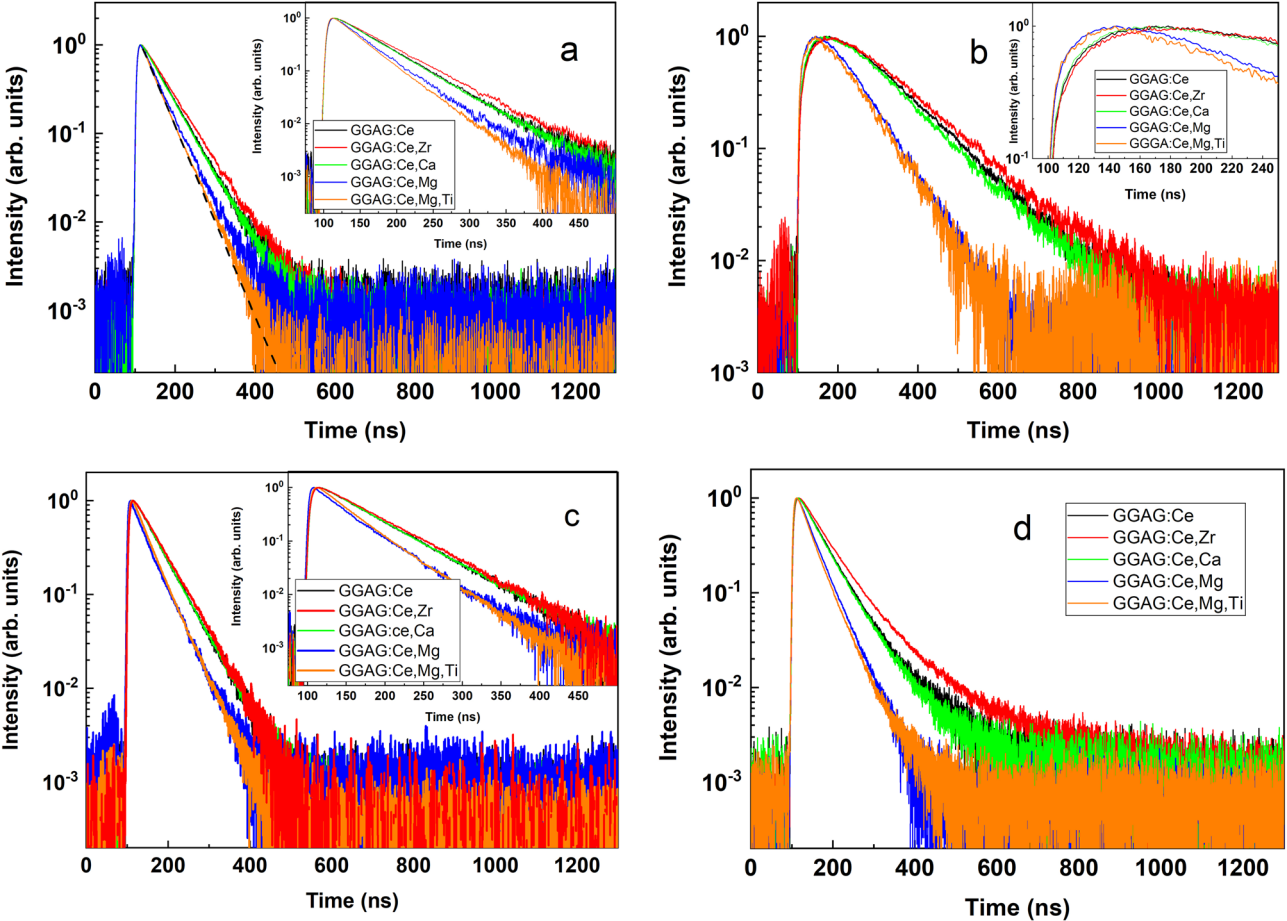
Luminescence decay kinetics of the Ce^3+^ emission (530 nm) in the co-doped GGAG:Ce single crystals under 450 nm (**a**), 275 nm (**b**), 235 nm (**c**) and 210 nm (**d**) excitations at room temperature. Black dashed line in (**a**) demonstrates an example of single exponential fit if the parameter of the decay time constant is 40 ns.

Figure 6 shows the decay kinetics of Ce^3+^ emission under tunable laser excitations of all studied GGAG crystals measured at room temperature. Four excitation wavelengths have been chosen: 450 nm, 275 nm, 235 nm and 210 nm. The 450 nm excitation corresponds to the *4f–5d*_*1*_ transitions in Ce^3+^ ion. Under 275 nm excitation the transition of ^8^S_7/2_ → ^6^I_J_ in Gd^3+^ ion takes place. Furthermore, the 235 nm excitation coincides with the excitation energy of the UV band, which we discussed above and preliminarily attributed to *4f–5d*_*3*_ transitions in Ce^3+^ ion. Finally, the 210 nm wavelength corresponds to the excitation of excitonic states in GGAG crystals. The arrows in the room temperature excitation spectrum in Fig. 3b indicate the last three excitation energies.

The decay kinetics of Ce^3+^ emission under direct excitation of Ce^3+^ ion via *4f–5d*_*1*_ transition (Fig. 6a) obey the single exponential law. However, decay time constant is different for different samples. The decay time constant for the decay kinetics observed in the Mg^2+^ and Mg + Ti co-doped crystals is about 40 ns, while for other crystals this parameter is about 60 ns. Please see the single exponential fit parameters in Figs. S2–S4 of the supplementary materials. We suggest that faster decay of luminescence in the Mg^2+^ and Mg + Ti co-doped crystals is due to partial non-radiative relaxation of Ce^3+^ ions near co-dopant impurities. Similar situation canbe observed in Fig. 6c under the 235 nm excitation. Again, the luminescence decay kinetics are faster for the Mg^2+^ and Mg + Ti co-doped crystals if to compare with other crystals, which reveal identical decay curves. Moreover, decay time constants obtained for each crystal is the same under the 450 nm and the 235 nm excitations. This fact confirms our suggestion above that the 235 nm (5.3 eV or UV) excitation band results from *4f–5d*_*3*_ transitions in Ce^3+^ ion.

The luminescence decay kinetics under the 275 nm excitation (Fig. 6b) are drastically different comparing with the considered above (Fig. 6a,c). Under the 275 nm excitation the transition ^8^S_7/2_ → ^6^I_J_ in Gd^3+^ ion occurs with further energy transfer to Ce^3+^ emission center.

The energy transfer mechanism from Gd^3+^ to Ce^3+^ is well established in other compounds^53–55^. However, the detailed mechanism of such energy transfer in GGAG:Ce has not been studied. Therefore, we suggest that under the 275 excitation of the ^8^S_7/2_ → ^6^I_J_ transition the subsequent non-radiative relaxation from ^6^I_J_ to the ^6^P_J_ level of Gd^3+^ takes place. Later on the relaxation from excited ^6^P_J_ level to ^8^S_7/2_ ground state leads either to radiative recombination of characteristic 317 nm emission of Gd^3+^ or energy transfers exiting the nearest Ce^3+^ ion. Taking into account that the ^6^P_J_-^8^S_7/2_ transition is forbidden electron’s lifetime on the ^6^P_J_ excited state is much longer than the decay time of Ce^3+^ emission. The population of ^6^P_J_ excited state of Gd^3+^ is fed from the ^6^I_J_ level and the ^6^I_J_-^6^P_J_ transition is also forbidden. Therefore, the energy transfer from Gd^3+^ to Ce^3+^ is limited by the population rate of ^6^P_J_, which is relatively slow. As a result, the decay kinetics of Ce^3+^ emission in Fig. 6b have slow rising fronts. These decay kinetics, in fact, represent superposition of the rise and decay of luminescence intensity. Nevertheless, one can see that the decay processes in the Mg^2+^ and Mg + Ti co-doped crystals occur faster than in other studied crystals. The faster decay in both magnesium co-doped crystals in Fig. 6b can be explained in the same way as we did before for the decay kinetics under the 450 nm and 235 nm excitations, i.e. by partial non-radiative relaxation of Ce^3+^ ions near co-dopant impurities. In respect of the decay kinetics under the excitation in Gd^3+^ absorption band, we should also note that the rise front of luminescence intensity observed in any single crystal in Fig. 6b has not been observed yet. For instance, in^56^ the decay kinetics of Ce^3+^ emission obtained in the GGAG:Ce and the Mg^2+^ co-doped GGAG:Ce crystals under the excitation in Gd^3+^ absorption band have not demonstrated any rise front of luminescence intensity at any temperature.

The luminescence decay kinetics excited by 210 nm photons (Fig. 6d) reveal deviation from single exponential decay, i.e. there are extra slow decay components in the decay kinetics. It is worth noting that the two-exponential decay fitting cannot properly approximate these decay kinetics. On the other hand, such fitting allows to evaluate roughly time constants of the slow decay component (see Figs. S5–S7 of the supplementary materials). However, main fast components of the decay kinetics in Fig. 6d are similar to those observed under the 450 nm and the 235 nm excitations. The slow components of the luminescence decay kinetics are connected with shallow traps in GGAG lattice. These traps did not influence the decay kinetics under other excitations (Fig. 6a–c) because only transitions within Ce^3+^ or Gd^3+^ ions were involved. The 210 nm excitation is capable to directly excite excitonic states in the GGAG lattice. Shallow traps of the crystal can trap charge carriers leading to the delay in Ce^3+^ radiative recombination. The strongest contribution of slow decay component is observed in the decay kinetics for the Zr^4+^ co-doped crystal. It means that this crystal contains a higher concentration of shallow traps among all crystals studied. We suggest that the centers, which compensate the extra positive charge induced by Zr^4+^ ions, can be effective traps for holes. On the other hand, the slow component in the luminescence decay kinetics of the Mg^2+^ and the Mg + Ti co-doped crystals is negligible small. This is in line with our suggestions analyzing the VUV excitation spectra (Fig. 4) that Mg^2+^ co-dopant ions suppress intrinsic defects in GGAG:Ce crystals.

Analyzing the decay kinetics of Ce^3+^ luminescence (Fig. 6a–d) we can conclude that decay kinetics are quite similar under any excitation for each sample. For instance, the decay kinetics observed in the Mg^2+^ co-doped crystal have the main component of the decay about 40 ns independently on the excitation energy. The slow rise front under the excitation in Gd^3+^ absorption band and the slow decay components under the excitation in the excitonic band are complimentary to the main decay component of 40 ns. The similar conclusion can be done also about the decay kinetics of other crystals studied. In our opinion, it is significant that the decay kinetics obtained for the Mg^2+^ and the Mg + Ti crystals are faster than the decay kinetics observed in other crystals under any low energy photo excitations. Decay kinetics of Ce^3+^ emission in the Mg^2+^ co-doped and non-co-doped GGAG:Ce single crystals have also been studied in^41^ under photo excitations in Ce^3+^ absorption bands. The decay kinetics obtained in^41^ were the same independently on the Mg^2+^ content and authors concluded that Mg^2+^ co-dopant does not noticeably perturb the energy levels of Ce^3+^. However, our results do not coincide with this conclusion and clearly show the influence of Mg^2+^ ions on the time evolution under excitation in the Ce^3+^, Gd^3+^ and excitonic absorption bands. This result may indicate that the faster scintillations in Mg^2+^ co-doped GGAG:Ce crystals is not only due to the fast formation of excited Ce^3+^ center from Ce^4+^ ion but also because of perturbed *5d* states of Ce^3+^ by Mg^2+^ co-dopants leading to the partial non-radiative relaxation. Similar conclusion was recently done in^57,58^ where the Mg^2+^ induced decrease of the scintillation light in GGAG:Ce films was explained by the overlapping of the lowest-energy excited *5d*_*1*_ level of Ce^3+^ and Mg^2+^ states.

## Conclusions

The luminescence properties of cerium doped as well as co-doped GGAG single crystals have been investigated by means of the vacuum ultraviolet (VUV) and soft X-ray (XUV) excitation spectroscopy utilizing synchrotron radiation and by means of time-resolved luminescence spectroscopy under tunable laser excitations. It was shown that GGAG:Ce single crystals having different co-dopant ions reveal distinguished efficiency of multiplication of electronic excitations in VUV spectral range. In addition, the strong influence of the annealing treatment GGAG:Ce single crystal on the Ce^3+^ luminescence excitation efficiency in VUV and XUV range was obtained. It was suggested that intrinsic defects in GGAG lattice are responsible for the capture of hot charge carriers leading to the degradation of the excitation efficiency in VUV and XUV range. It is found that the best efficiency of multiplication of electronic excitation processes in VUV and XUV range demonstrated in the Mg^2+^ co-doped GGAG:Ce single crystal. It was also shown that the annealing in vacuum can drastically improve performance of GGAG:Ce crystal increasing luminescence efficiency in VUV and XUV excitation region. It is suggested that the Mg^2+^ co-doping or the annealing in vacuum strongly suppresses intrinsic defects in GGAG:Ce single crystals removing competing relaxation channels of hot charge carriers.

The combination of time-resolved luminescence spectroscopy and excitation spectroscopy techniques allows to conclude that the excitation band of Ce^3+^ emission peaking at about 235 nm (5.3 eV) is due to *4f–5d*_*3*_ transitions in Ce^3+^ ion in GGAG crystals. It was observed that Ce^3+^ luminescence decay is faster in the Mg^2+^ co-doped crystals under any photo excitations below the energy of band-to-band transitions in the GGAG. It means that improved scintillating properties of Mg^2+^ co-doped GGAG:Ce crystals known in literature are driven not only by the formation of luminescence center from Ce^4+^ ion but also by the perturbation of Ce^3+^ ion by Mg^2+^ co-dopants.

## Supplementary information

Supplementary Information.
