# Supplementary material for: Time-resolved luminescence and excitation spectroscopy of Co-doped Gd3Ga3Al2O12 scintillating crystals
Source: Sci Rep. 2020 Nov 23;10:20388. doi: 10.1038/s41598-020-77451-x (PMC7684308; doi:10.1038/s41598-020-77451-x)
Supplement: Supplementary file 1 — Supplementary Information. [file 41598_2020_77451_MOESM1_ESM.pdf]

Supplementary material

Time-Resolved Luminescence and Excitation Spectroscopy of Co-Doped $Gd_3Ga_3Al_2O_{12}$ Scintillating Crystals

Viktorija Pankratova[1], Anna P. Kozlova[2], Oleg A. Buzanov[3], Kirill Chernenko[4], Roman Shendrik[5], Anatolijs Šarakovskis[1] and Vladimir Pankratov[1,2] (corresponding author's email: vladimirs.pankratovs@cfi.lu.lv)

[1]*Institute of Solid State Physics, University of Latvia, 8 Kengaraga iela, LV-1063 Riga, Latvia*

[2]*National University of Science and Technology "MISiS", Leninsky Prospekt 4, 119049 Moscow, Russia*

[3]*OJSC "Fomos-Materials" Co., Buzheninova street 16, 107023 Moscow, Russia*

[4]*MAX IV Laboratory, Lund University, PO BOX 118, SE-221 00 Lund, Sweden*

[5]*Vinogradov Institute of Geochemistry, SB RAS, 1a Favorskii Street, 664033 Irkutsk, Russia*

1. **The calculations of the attenuation length of photons 30 eV-1000 eV**

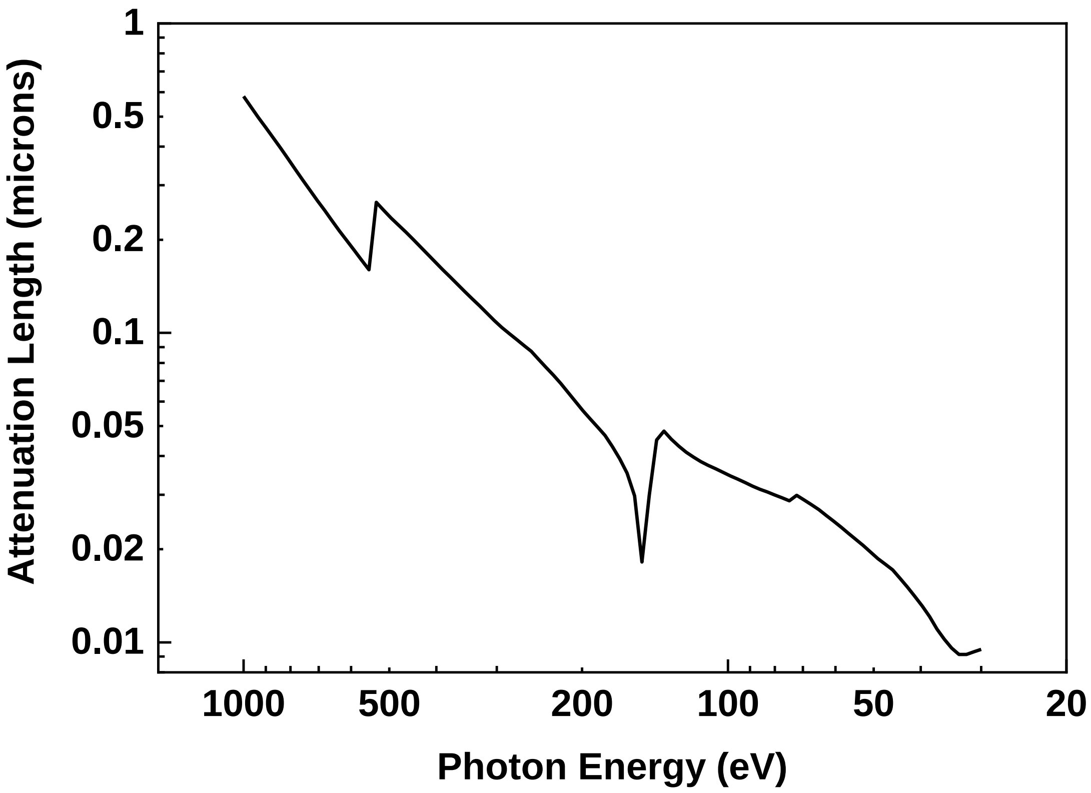

Fig. S1. The calculated attenuation length of photons 30 eV-1000 eV falling on the GGAG (g=6.63 g/cm3). The calculations have been done using following resource: https://henke.lbl.gov/optical_constants/atten2.html

2. Exponential fit of the decay kinetics using Origin commercial software.

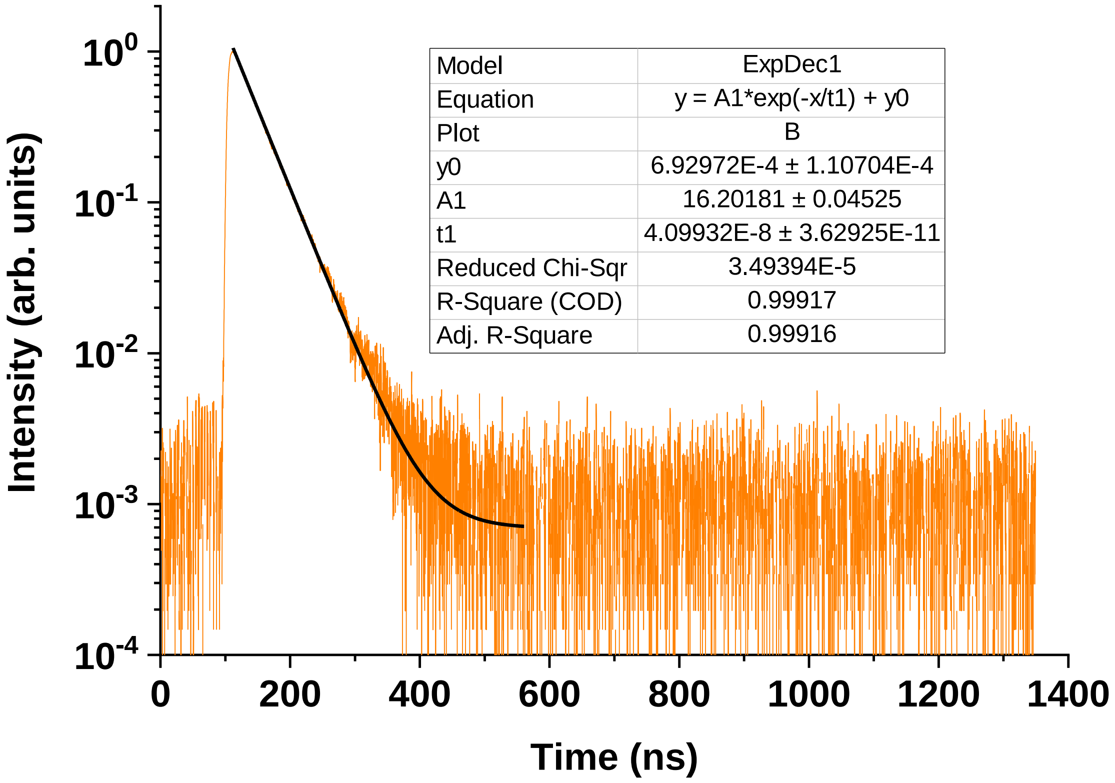

Fig. S2. The single exponential fit of the Ce^{3+} luminescence decay kinetic in the GGGA:Ce single crystal co-doped by Mg+Ti taken from Fig. 6a. The curve fit was done by standard commercial Origin software. Fit parameters are summarized in the table inset.

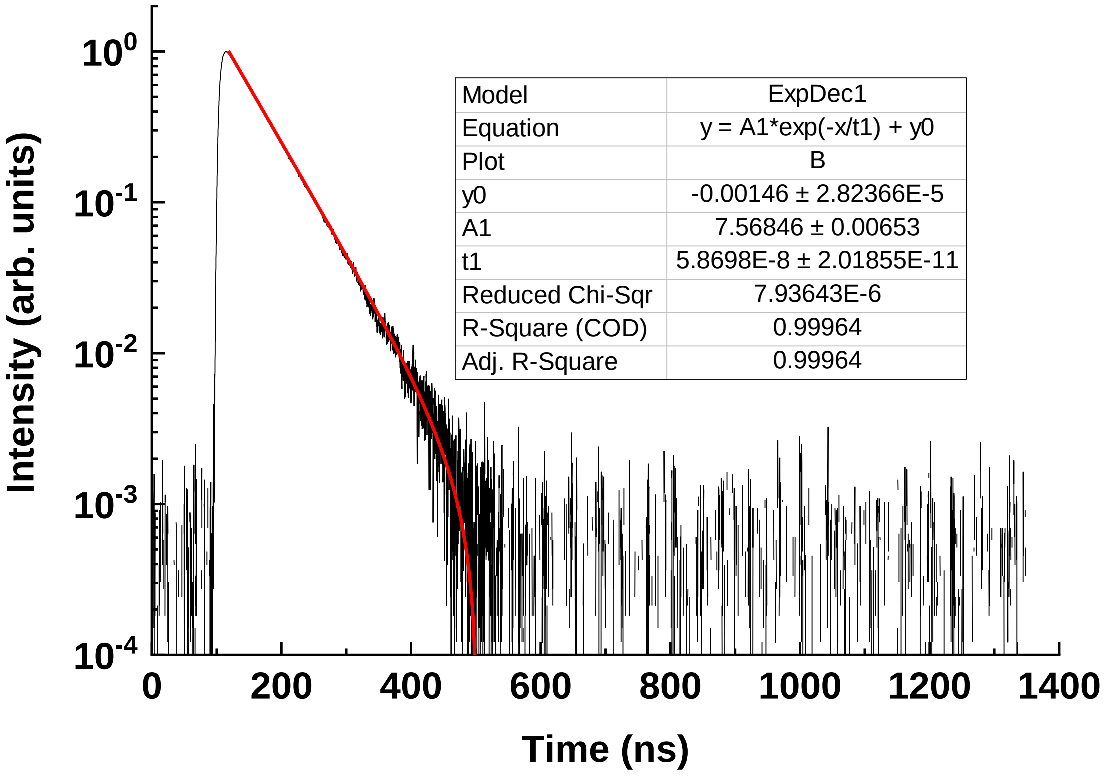

Fig. S3. The single exponential fit of the Ce^{3+} luminescence decay kinetic in the non-codoped GGGA:Ce single crystal taken from Fig. 6a. The curve fit was done by standard commercial Origin software. Fit parameters are summarized in the table inset.

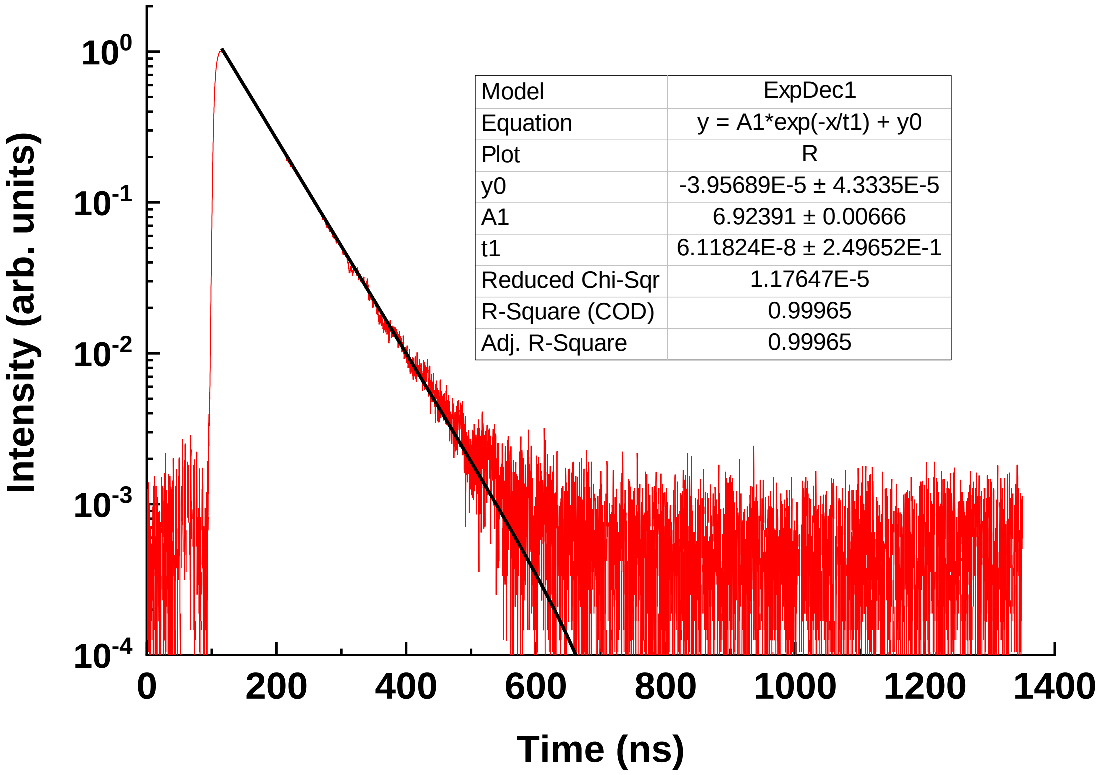

Fig. S4. The single exponential fit of the Ce^{3+} luminescence decay kinetic in the GGGA:Ce single crystal co-doped by Zr taken from Fig. 6a. The curve fit was done by standard commercial Origin software. Fit parameters are summarized in the table inset.

3. Two-exponential fit of the decay kinetics using Origin commercial software.

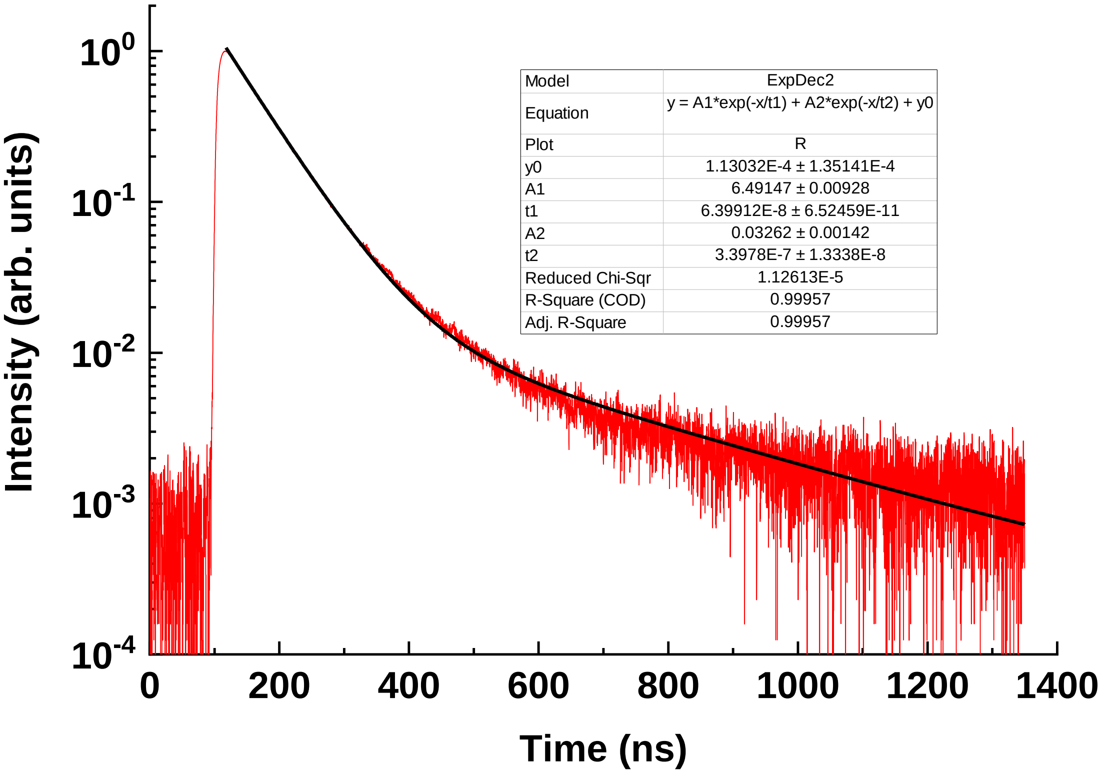

Fig. S5. The two-exponential fit of the Ce^{3+} luminescence decay kinetic in the Zr^{4+} codoped GGGA:Ce single crystal taken from Fig. 6d. The curve fit was done by standard commercial Origin software. Fit parameters are summarized in the table inset.

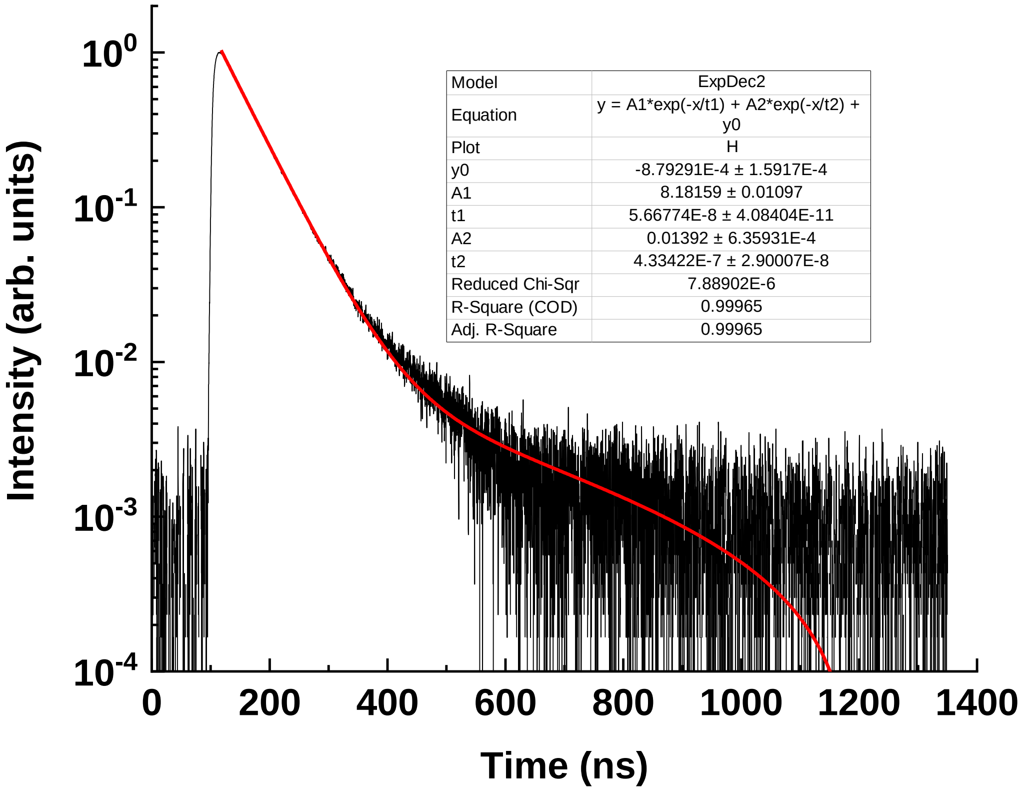

Fig. S6. The two-exponential fit of the Ce^{3+} luminescence decay kinetic in the non-codoped GGGA:Ce single crystal taken from Fig. 6d. The curve fit was done by standard commercial Origin software. Fit parameters are summarized in the table inset.

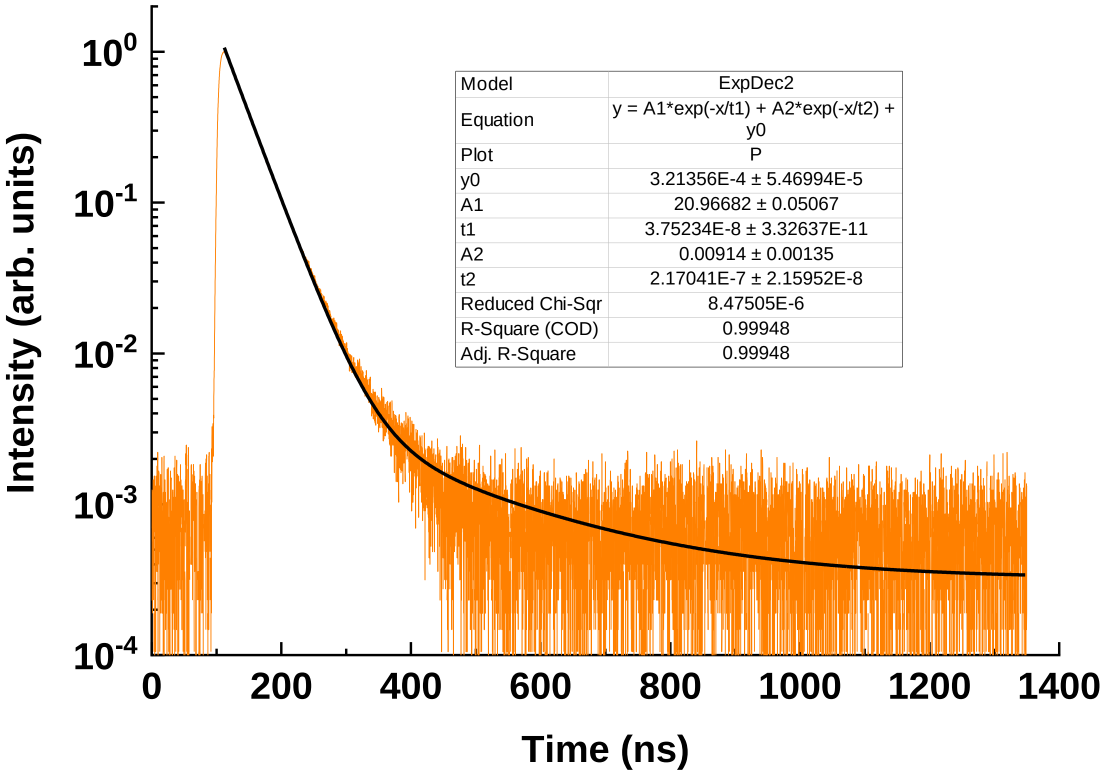

Fig. S7. The two-exponential fit of the Ce^{3+} luminescence decay kinetic in the Mg+Ti codoped GGGA:Ce single crystal taken from Fig. 6d. The curve fit was done by standard commercial Origin software. Fit parameters are summarized in the table inset.
